# Hes1 marks peri-condensation mesenchymal cells that generate both chondrocytes and perichondrial cells in early bone development

**DOI:** 10.1016/j.jbc.2023.104805

**Published:** 2023-05-11

**Authors:** Yuki Matsushita, Hiroaki Manabe, Takahiro Ohyama, Shogo Nakamura, Mizuki Nagata, Wanida Ono, Noriaki Ono

**Affiliations:** 1 Department of Diagnostic and Biomedical Sciences, University of Texas Health Science Center at Houston School of Dentistry, Houston, Texas, USA; 2 Department of Cell Biology, Nagasaki University Graduate School of Biomedical Sciences, Nagasaki, Japan

**Keywords:** Hes1, Notch signaling, mesenchymal condensation, perichondrium, cartilage, perichondrial cells, chondrocytes, osteoblasts, in vivo lineage-tracing, single-cell RNA-sequencing, endochondral bone development

## Abstract

Bone development starts with condensations of undifferentiated mesenchymal cells that set a framework for future bones within the primordium. In the endochondral pathway, mesenchymal cells inside the condensation differentiate into chondrocytes and perichondrial cells in a SOX9-dependent mechanism. However, the identity of mesenchymal cells outside the condensation and how they participate in developing bones remain undefined. Here we show that mesenchymal cells surrounding the condensation contribute to both cartilage and perichondrium, robustly generating chondrocytes, osteoblasts, and marrow stromal cells in developing bones. Single-cell RNA-seq analysis of *Prrx1-cre*-marked limb bud mesenchymal cells at E11.5 reveals that Notch effector *Hes1* is expressed in a mutually exclusive manner with *Sox9* that is expressed in pre-cartilaginous condensations. Analysis of a Notch signaling reporter *CBF1:H2B-Venus* reveals that peri-condensation mesenchymal cells are active for Notch signaling. *In vivo* lineage-tracing analysis using *Hes1-creER* identifies that Hes1^+^ early mesenchymal cells surrounding the SOX9^+^ condensation at E10.5 contribute to both cartilage and perichondrium at E13.5, subsequently becoming growth plate chondrocytes, osteoblasts of trabecular and cortical bones, and marrow stromal cells in postnatal bones. In contrast, Hes1^+^ cells in the perichondrium at E12.5 or E14.5 do not generate chondrocytes within cartilage, contributing to osteoblasts and marrow stromal cells only through the perichondrial route. Therefore, Hes1^+^ peri-condensation mesenchymal cells give rise to cells of the skeletal lineage through cartilage-dependent and independent pathways, supporting the theory that early mesenchymal cells outside the condensation also play important roles in early bone development.

Bone development involves highly sequential steps of mesenchymal cell proliferation and differentiation, thus represents a prime example of organogenesis deliberately executed through heterotypic cellular interactions. Condensation of undifferentiated mesenchymal cells is the first step of bone development, which sets a framework for future bones within the primordium (1, 2). In this process, a previously dispersed population of undifferentiated mesenchymal cells migrate to the site of the future skeleton in the limb bud and aggregate to form a vasculature-free element termed mesenchymal condensation (3). Mesenchymal condensations serve as an important modality to generate skeletal precursor cells on a large scale in a highly organized environment. Particularly in the endochondral pathway by which most of mammalian bones are formed, mesenchymal condensations give rise to both cartilage template and perichondrium in the subsequent phase. Chondrocytes within the cartilage template and perichondrial cells provide distinct cellular sources of growth plate chondrocytes, trabecular and cortical osteoblasts, and marrow stromal cells (4, 5, 6, 7). Therefore, mesenchymal condensations provide the foundation of developing bones.

The current concept holds that SOX9, a master transcription factor for chondrogenesis, is essential for condensing mesenchymal cells to differentiate into chondrocytes and perichondrial cells (1, 8, 9, 10). Indeed, SOX9 is absolutely required for these mesenchymal cells to stay organized within the condensation (11). Fate-mapping studies using *Sox9-cre/creER* demonstrate that Sox9^+^ cells within the condensation function as osteo-chondro-progenitor cells as they give rise to chondrocytes and perichondrial cells in the subsequent stage (8, 9, 12). In the limb, *Prrx1-cre* is used to mark the entire spectrum of cells of the skeletal lineage including “skeletal stem cells” and their derivatives, as *Prrx1* is expressed not only by condensing mesenchymal cells but also by other cells that originate from the lateral plate mesoderm (13, 14). Interestingly, *Prrx1* is predominantly expressed in the perichondrium at E13.5 when the cartilage template is established (4, 15, 16), suggesting that cells surrounding the condensation may also robustly contribute to the generation of skeletal lineage cells. However, the identity of early mesenchymal cells outside the condensation and how they participate in developing bones remain undefined.

We recently demonstrated that Dlx5^+^ early perichondrial cells located in the outer layer of the perichondrium at E12.5 sustainably contribute to cortical bone and marrow stromal compartments in developing bones (4). These Dlx5^+^ perichondrial cells remain in the perichondrium in subsequent stages, unlike osterix (Osx^+^) osteogenic perichodrial cells that are in the inner layer of the perichondrium (17). Osx^+^ cell descendants contribute to skeletogenesis only transiently and eventually disappear from the perichondrium and the skeletal element (8, 18). Interestingly, however, Dlx5^+^ early perichondrial cells do not contribute to cartilage. It has not been determined if peri-condensation mesenchymal cells preceding Dlx5^+^ perichondrial cells can generate chondrocytes within the cartilage template.

In this study, we hypothesize that early mesenchymal cells surrounding the condensation can contribute to chondrocytes of the cartilage template while also remaining outside the condensation and contributing to perichondrial cells. To test this hypothesis, we utilized *in vivo* lineage-tracing approaches to define the cell fates of peri-condensation mesenchymal cells. Using single-cell RNA-seq analyses, we identified Notch effector *Hes1* as a potential marker of peri-condensation mesenchymal cells that are located outside the SOX9^+^ condensation and subsequently revealed their cell fates using a tamoxifen-inducible *Hes1-creER* allele. Our findings demonstrate that Hes1^+^ peri-condensation mesenchymal cells give rise to cells of the skeletal cell lineage through cartilage-dependent and independent pathways in growing bones and critically support early bone development.

## Results

### Single-cell RNA-seq identifies Hes1 as a potential marker of peri-condensation mesenchymal cells

**Figure 1:**
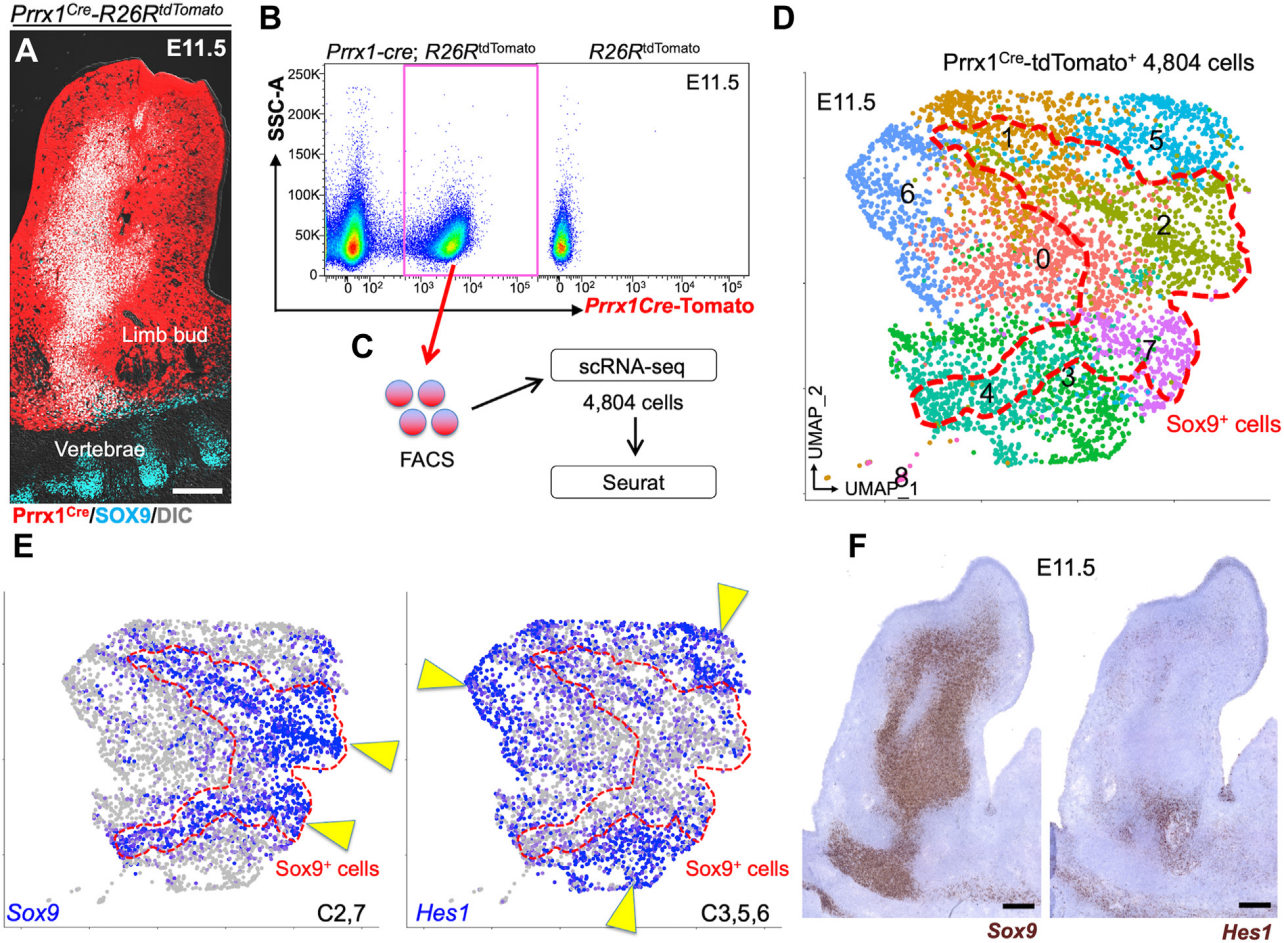
**Single cell RNA-seq reveals the heterogeneity of early mesenchymal cells in the limb bud.***A*–*E*, Single cell RNA-seq analysis of *Prrx1-cre*-marked limb bud mesenchymal cells at E11.5. *A*, *Prrx1-cre*; *R26R*^tdTomato^ femur stained for SOX9. Scale bar: 200 μm. *n* = 3 mice. *B* and *C*, FACS (*B*) and scRNA-seq (*C*) strategy for Prrx1^cre^-tdTomato^+^ cells (*red box*). Shown are cells isolated from *Prrx1-cre*; *R26R*^tdTomato^ limb buds (*left*) or *R26R*^tdTomato^ control limb buds (*right*). *D*, UMAP-based visualization of major classes of Prrx1^cre^-tdTomato^+^ cells (Cluster 0–8). *E*, feature plots. *Blue*: high expression. *Arrowheads*: clusters in which a given gene is identified as a cell type-specific marker. Cluster 2,7: *Sox9*^+^, Cluster 3,5,6: *Hes1*^+^. Dotted contour: Sox9^+^ cells. *n* = 4804 cells. Pooled from *n* = 5 mice. *F*, RNAscope *in situ* hybridization analysis of E11.5 limb bud for *Hes1* and *Sox9* mRNA. Scale bar: 200 μm. *n* = 3 mice.

In the mouse limb bud, undifferentiated mesenchymal cells make condensations around embryonic day (E) 10.5. These condensing mesenchymal cells express SOX9 to activate the transcription machinery for chondrocyte differentiation (19). We first performed single-cell RNA-seq analyses to define cellular heterogeneity of the limb bud mesenchyme. Limb bud mesenchymal cells contributing to the skeletal element, including SOX9^+^ condensing mesenchymal cells are ubiquitously marked by a *cre* recombinase driven by a 2.4kb *Prrx1* promoter/enhancer (13) (Fig. 1*A*). We dissociated limb mesenchymal cells of *Prrx1-cre*, *R26R*^tdTomato^ mice at embryonic day 11.5 (E11.5), and isolated tdTomato^+^ cells by fluorescence-activated cell sorting (FACS) (Fig. 1*B*). We profiled 4804 cells Prrx1^cre^-tdTomato^+^ cells using the 10X Chromium Single-Cell Gene Expression Solution platform (Fig. 1*C*). A graph-based clustering analysis using Seurat (20) revealed 9 clusters (Fig. 1*D*), including two clusters of cells abundant in *Sox9* (Cluster 2,7, arrowheads of Fig. 1*E* left) and six clusters of their surrounding cells (Cluster 0,1,3–6). *Prrx1* was expressed in the surrounding cluster (Cluster 5) while another chondrogenic marker *Col2a1* expression overlapped with *Sox9* expression (cluster 2,7) (Fig. S1*A*). Notably, *Sox9* and *Col2a1* were also expressed in a part of clusters 1, 3, and 4 (red dotted contour of Fig. 1, *D* and *E*). The surrounding clusters were composed of mesenchymal cells expressing unique homeobox proteins, such as *Msx1* (Cluster 5,6), *Lhx9* (Cluster 5), *Meox2* (Cluster 0), *Emx2* (Cluster 4) and *Irx3/5* (Cluster 3,7) (Fig. S1*A*). In addition, *Shh* was expressed in Cluster 6, indicating that this cluster corresponds to cells on the posterior margin of the limb bud (21). Therefore, the limb bud skeletal element is constituted by distinct groups of mesenchymal cells constituting the SOX9^+^ condensation and its surrounding structures.

In search for a marker for peri-condensation mesenchymal cells, we noticed that a Notch effector gene *Hes1* exhibited a pattern negatively correlated with *Sox9*, identified as cell-type specific markers for Cluster 3,5,6 (Fig. 1*E*). Notch signaling is essential for maintaining skeletal progenitor cells, and its target gene *Hes1* is most abundantly expressed in the fetal perichondrium (22, 23). RNAscope analysis revealed that *Hes1* was expressed in a manner surrounding the *Sox9*-expressing condensation (Fig. 1*F*). To further validate Notch-responsive status of peri-condensation mesenchymal cells *in situ*, we utilized a Notch signaling reporter strain expressing a histone 2B (H2B)-bound Venus protein under a *C promoter binding factor 1 (CBF1)* promoter (*CBF1:H2B-Venus*) (24). Interestingly, Notch-responsive Venus^bright^ cells were mostly located outside the SOX9^+^ domain of the condensation (Fig. S1*B*). Therefore, Notch signaling is active in mesenchymal cells surrounding the SOX9^+^ condensation. In subsequent *in vivo* lineage-tracing studies, we focused on Notch effector *Hes1* as a marker of peri-condensation mesenchymal cells.

### Hes1-creER^+^ peri-condensation mesenchymal cells can generate chondrocytes

**Figure 2:**
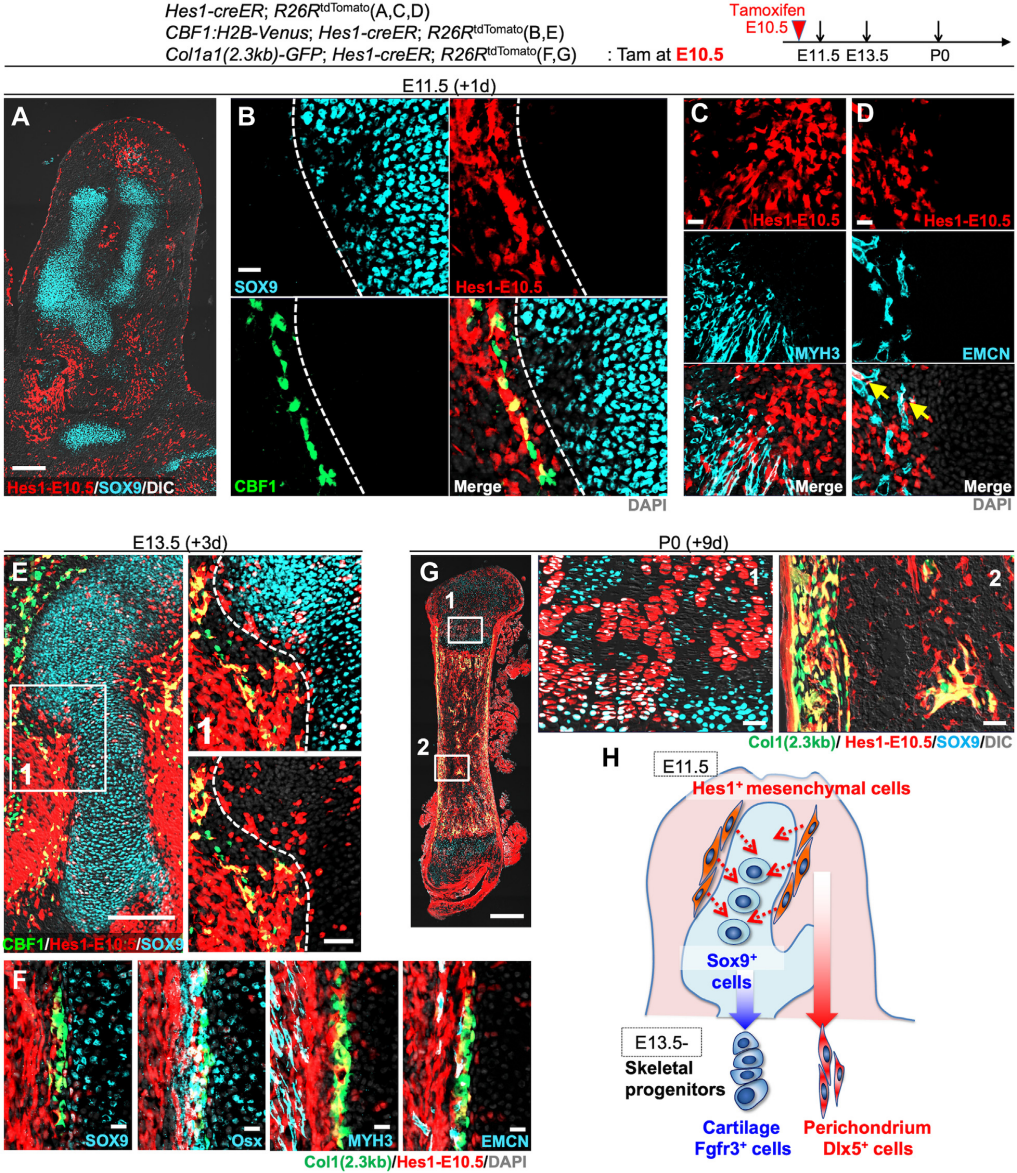
**Undifferentiated Hes1-creER**^**+**^**cells surrounding the mesenchymal condensation provide skeletal progenitor cells.***A*–*G*, Cell-fate analysis of *Hes1-creER*^+^ mesenchymal cells of the condensation stage, pulsed at E10.5. *Hes1-creER*; *R26R*^tdTomato^ femurs carrying *CBF1*:*Venus* (*B* and *E*) or *Col1a1(2.3kb)-GFP* (*F* and *G*) reporters. *A*, Limb bud at E11.5. Scale bar: 200 μm. *n* = 4 mice. *B*, Magnified view of mesenchymal condensation at E11.5. Scale bar: 20 μm. *n* = 4 mice. *C* and *D*, Immunostaining for MYH3 (*C*) and endomucin (EMCN) (*D*). *Arrow*: *Hes1-creER*^+^Emcn^+^ cells. Scale bar: 20 μm. *n* = 4 mice. *E*, Cartilage template at E13.5. *Right panel*: magnified view of Area (1). Dotted line: cartilage-perichondrium border. *Grey*: DIC. Scale bar: 200 μm (*left*), 50 μm (*right*). *n* = 4 mice. *F*, Cartilage-perichondrium immunostaining for SOX9, osterix (OSX), MYH3 and EMCN. Scale bar: 20 μm. *n* = 4 mice. *G*, Neonatal femur at P0, after 9 days of chase. *Right panels*: magnified views of (1: growth plate, 2: bone marrow). Scale bar: 200 μm (*left*), 20 μm (right 2 panels). *n* = 4 mice. *H*, Diagram of Hes1^+^ mesenchymal cell fates. Hes1^+^So x 9^neg^ cells surrounding the condensation are *bona fide* skeletal progenitor cells during endochondral bone development, contributing to both chondrocytes and perichondrial cells and subsequently to all limb skeletal cells. MYH3, myosin heavy chain 3.

To study the cell fates of these Hes1^+^ cells surrounding Sox9^+^ condensation, we performed lineage-tracing experiments using a *Hes1-creER* knock-in allele (25) that activates an *R26R*-tdTomato reporter in a tamoxifen-dependent manner. First, we pulsed *Hes1-creER*; *R26R*^tdTomato^ mice at E10.5 and analyzed these mice after 24 h at E11.5 to define the identities of *Hes1-creER*^+^ cells (Hes1^CE^-E10.5 cells). Hes1^CE^-E10.5 cells were located throughout the limb bud at E11.5 in a manner excluding the SOX9^+^ pre-cartilaginous condensation (Fig. 2*A*). Hes1^CE^-E10.5 cells were also located in the area immediately adjacent to SOX9^+^ cells associated with CBF1:H2B-Venus reporter activities (Fig. 2*B*). We also noticed that *Hes1-creER* simultaneously marked other types of cells outside the skeletal elements of the limb bud, including myosin heavy chain 3 (MYH3^+^) skeletal muscle cells and endomucin (EMCN^+^) endothelial cells (Fig. 2, *C* and *D*). Therefore, *Hes1-creER* can mark mesenchymal cells in the SOX9-negative domain of the condensation upon tamoxifen injection.

Subsequently, we traced the fate of Hes1-creER^+^ cells in endochondral bone development (see Fig. S2, *A* and *B* for H&E and Alcian Blue staining of developing endochondral bones). After 3 days of chase at E13.5, Hes1^CE^-E10.5 cells contributed to both chondrocytes and perichondrial cells of the cartilage template at E13.5 (Fig. 2*E*). Hes1^CE^-E10.5 cells became SOX9^+^ chondrocytes within the cartilage template while contributing to a majority of perichondrial cells including those expressing CBF1:H2B-Venus (Fig. 2*E*, right panel). In fact, Hes1^CE^-E10.5 cells contributed to all layers of the perichondrium including OSX^+^ osteoblast precursors, in addition to MYH3^+^ skeletal muscle cells outside the perichondrium and EMCN^+^ endothelial cells (Figs. 2*F* and S3*A*). After 9 days of the chase at postnatal day (P) 0, Hes1^CE^-E10.5 cells contributed to many columnar chondrocytes of the growth plate, Col1a1-GFP^+^ osteoblasts on the cortical and trabecular bone, and stromal cells throughout the marrow space (Fig. 2*G*). We also examined the cell fate of Hes1^+^ cells at an earlier stage. For this purpose, we pulsed *Hes1-creER; R26R*^tdTomato^ mice at E8.5 and analyzed these mice at E13.5 and E18.5. Hes1^CE^-E8.5 cells contributed to essentially all skeletal cells, robustly generating chondrocytes, perichondrial cells, osteoblasts, and marrow stromal cells (Fig. S3, *C* and *D*). Notably, Hes1^CE^-E8.5 cells contributed more substantially to the skeletal lineage than Hes1^CE^-E10.5 cells did, indicating that Hes1^+^ cells at a pre-condensation stage might possess a strong chondrogenic potential.

Importantly, skeletal muscle cells or endothelial cells that were initially marked by *Hes1-creER* at E10.5 did not contribute to these skeletal lineage cells, as cells marked by skeletal muscle-specific *cre* lines (*Acta1-cre*, *Myl1-cre*, and *Mck-cre*) or an endothelial cell-specific *cre* line (*Tie2-cre*) did not contribute to chondrocytes, osteoblasts, or marrow stromal cells at any time points (Fig. S4, *A* and *B*). This allows us to exclude the contribution of skeletal muscle cells and endothelial cells initially labelled by *Hes1-creER* at E10.5 to skeletal lineage cells.

Therefore, Hes1^+^ mesenchymal cells surrounding the condensation can differentiate not only into perichondrial cells but also into chondrocytes within the cartilage template, both of which robustly contribute to chondrocytes, osteoblasts, and marrow stromal cells in the later stages of endochondral bone development (Fig. 2*H*).

### Hes1-creER^+^ perichondrial cells possess osteogenic but not chondrogenic potential

Subsequently, we sought to define the cell fate of Hes1^+^ cells in the later stages of endochondral bone development, at E12.5 when the perichondrium is established adjacent to the cartilage template, as well as at E14.5 when the formation of the marrow space starts due to vascular invasion into the cartilage template.

**Figure 3:**
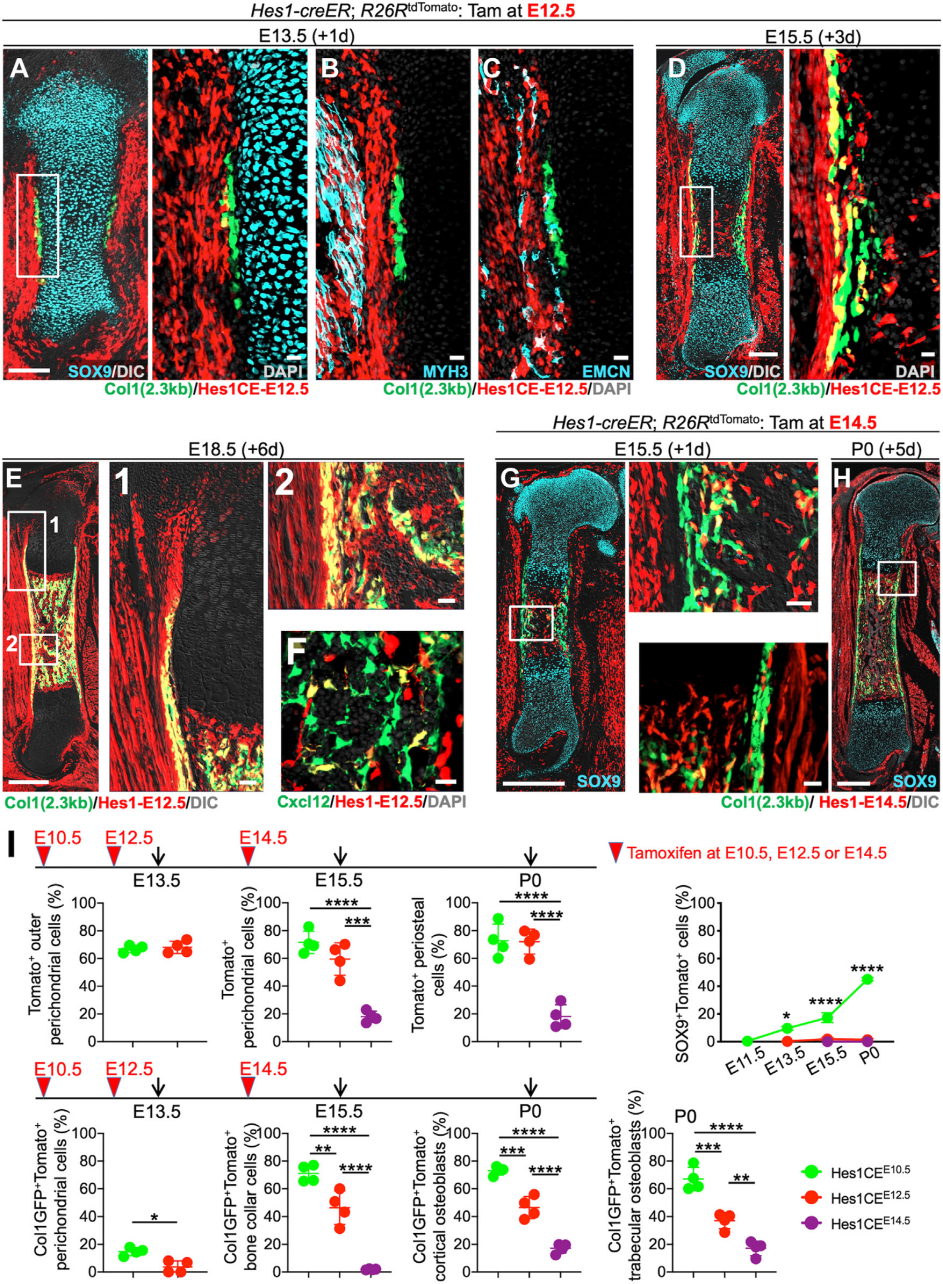
**Hes1-creER**^**+**^**cells provide fewer skeletal progenitor cells at becoming later embryonic stages.***A*–*F*, Cell-fate analysis of *Hes1-creER*^+^ cells, pulsed at E12.5, carrying *Col1a1(2.3kb)-GFP* reporters. *A*–*C*, Cartilage template at E13.5. Immunostaining for SOX9 (*A*), MYH3 (*B*), and EMCN (C). Right panel of (*A*): magnified view of the boxed area. Scale bar: 200 μm (*A*-left). 20 μm (*A*-right, *B* and *C*). *n* = 4 mice. *D*, *Left panel*: whole femur at E15.5. Scale bar: 200 μm. *Right panel*: magnified view of the boxed area. *n* = 4 mice. Scale bar: 20 μm. *E*, Whole femur at E18.5. *Right panels*: magnified view of the boxed area (1, 2). Perichondrium, bone collar and growth plate. Scale bar: 500 μm (*left*), 50 μm (right 2 panels). *n* = 4 mice. *F*, Bone marrow at E18.5. Scale bar: 20 μm. *n* = 3 mice. *G* and *H*, Cell-fate analysis of *Hes1-creER*^+^ cells, pulsed at E14.5, carrying *Col1a1(2.3kb)-GFP* reporters. *G*, Whole femur at E15.5. *Right panel*: magnified view of the boxed area. Scale bar: 500 μm (*left*), 50 μm (*right*). *n* = 4 mice. (H) Whole femur at E15.5. *Left panel*: magnified view of the boxed area. Scale bar: 500 μm (*right*), 50 μm (*left*). *n* = 4 mice. *I*, Quantitative analysis of Hes1-E10.5-, Hes1-E12.5- or Hes1-E14.5-tdTomato^+^ cells’ contribution to skeletons in embryonic and neonatal stages. *Green*: Hes1-E10.5, *Red*: Hes1-E12.5, Violet: Hes1-E14.5. *Upper left* three panels: Percentage of total tdTomato^+^ outer perichondrial cells among total outer perichondrial cells at E13.5 (leftmost) and E15.5 (second from the left), and tdTomato^+^ periosteal cells among total periosteal cells at P0 (third from the left). *n* = 4 mice per each group. Upper rightmost panel: Contribution of *Hes1-creER*^+^ (E10.5, E12.5 and E14.5) cells to SOX9^+^ chondrocytes within the cartilage template. *n* = 4 mice per each group. Lower panels: Percentage of Col1a1(2.3kb)-GFP^+^tdTomato^+^ cells among total Col1a1(2.3kb)-GFP^+^ cells at E13.5 (inner perichondrium, leftmost), E15.5 (bone collar, second from the left) and P0 (cortical bone, third from the left. Trabecular bone, rightmost). *n* = 4 mice per each group. ∗*p* < 0.05, ∗∗*p* < 0.01, ∗∗∗*p* < 0.001, ∗∗∗∗*p* < 0.0001. Two-tailed, Mann–Whitney *U* test (upper and lower leftmost panels). Two-tailed, One-way ANOVA followed by Tukey’s *post hoc* test (the others). Data are presented as mean ± SD. MYH3, myosin heavy chain 3.

To this end, we first pulsed *Col1a1(2.3kb)-GFP*; *Hes1-creER*; *R26R*^*tdTomato*^ mice at E12.5 and analyzed these mice after 24 h at E13.5 to define the characteristics of *Hes1-creER*^*+*^ cells in the perichondrium (Hes1^CE^-E12.5 cells). Hes1^CE^-E12.5 cells were located outside the SOX9^+^ domain of the cartilage template with minimal overlap with Col1a1-GFP^+^ osteoblasts in the perichondrium (Fig. 3*A*). Hes1^CE^-E12.5 cells were also found outside the skeletal element among MYH3^+^ skeletal muscle cells and EMCN^+^ endothelial cells (Fig. 3, *B* and *C*).

We subsequently traced the fate of Hes1^CE^-E12.5 perichondrial cells during the formation of the primary ossification center. After 3 days of chase at E15.5, Hes1^CE^-E12.5 cells contributed to cells within the primary ossification center as well as Col1a1-GFP^+^ osteoblasts in the bone collar (Fig. 3*D*). After 6 days of chase at E18.5, Hes1^CE^-E12.5 cells contributed to Cxcl12-GFP^high^ marrow stromal cells and Col1a1-GFP^+^ osteoblasts in the trabecular bone (Fig. 3, *E* and *F*). Importantly, unlike those pulsed at an earlier stage, Hes1^CE^-E12.5 cells did not contribute to SOX9^+^ chondrocytes in the growth plate but maintained themselves within the perichondrium, and robustly contributed to the bone collar and Col1a1-GFP^+^ osteoblasts in the cortical bone (Fig. 3*E*). Therefore, *Hes1-creER* marks perichondrial cells with robust capability to contribute to cortical and trabecular bone compartments as well as to marrow stromal compartments, akin to those marked by *Dlx5-creER* that we recently reported (4).

We also traced the fate of Hes1-creER^*+*^ cells at E14.5 (Hes1^CE^-E14.5 cells). At E15.5, Hes1^CE^-E14.5 cells were located among the perichondrium, the periosteum, and the primary ossification center, although most of these cells appeared to be non-skeletal with minimal overlap with Col1a1-GFP^+^ osteoblasts (Fig. 3*G*). After 5 days of chase at P0, Hes1^CE^-E14.5 cells did not robustly contribute to Col1a1-GFP^+^ osteoblasts in the cortical and trabecular bones (Fig. 3*H*), indicating that Hes1^+^ cells lose robust osteogenic potential at E14.5. Postnatally, Hes1-creER^+^ cells at P3 (Hes1^CE^-P3 cells) were localized in the groove of Ranvier, the metaphyseal primary spongiosa and the superficial layer of the articular cartilage at P5 (Fig. S5). Hes1^CE^-P3 cells did not overtly overlap with SOX9^+^ cells within the growth plate, whereas a few Hes1^CE^-P3 cells in a deeper layer of the articular cartilage expressed SOX9. Therefore, Hes1^+^ cells do not overlap with SOX9^+^ cells at a later stage of perichondrial development.

We further performed quantitative histological approaches to determine how Hes1^+^ cells at different stages (Hes1^CE^-E10.5, Hes1^CE^-E12.5, and Hes1^CE^-E14.5 cells) differentially contribute to endochondral bone development. First, we quantified the contribution of Hes1^CE^-E10.5 or Hes1^CE^-E12.5 cells to the E13.5 perichondrium among outer Col1a1-GFP^neg^ perichondrial cells (top) or inner Col1a1-GFP^+^ osteogenic perichondrial cells (bottom). Hes1^+^ peri-condensation mesenchymal cells robustly contributed to osteogenic perichondrial cells, as Hes1^CE^-E10.5 cells contributed to a significantly higher fraction of Col1a1-GFP^+^ osteogenic perichondrial cells, while both Hes1^CE^-E10.5 and Hes1^CE^-E12.5 cells constituted an equivalent fraction of outer perichondrial cells (Fig. 3*I*, first panels from the left).

Second, we quantified the contribution of Hes1^CE^-E10.5, Hes1^CE^-E12.5 or Hes1^CE^-E14.5 cells to the E15.5 perichondrium and bone collar, among outer Col1a1-GFP^neg^ perichondrial cells (top) or inner Col1a1-GFP^+^ osteogenic bone collar cells (bottom). *Hes1-creER* did not effectively mark perichondrial cells at E14.5, as Hes1^CE^-E14.5 cells contributed to a significantly fewer fraction of outer perichondrial cells than Hes1^CE^-E10.5 or Hes1^CE^-E12.5 cells did (Fig. 3*I*, second panel from the left in the upper panels). Hes1^CE^-E10.5 cells contributed to a majority of Col1a1-GFP^+^ osteogenic cells in the bone collar. However, the osteogenic potential of Hes1^+^ cells declined in later stages, as Hes1^CE^-E12.5 and Hes1^CE^-E14.5 cells contributed to progressively fewer fractions of inner Col1a1-GFP^+^ osteogenic cells in the bone collar (Fig. 3*I*, second panel from the left in the lower panels). These trends continued onto P0, as the contribution of Hes1^+^ cells to cortical and trabecular bone osteoblasts progressively declined in later stages (Fig. 3*I*, third panel from the left in the lower panels).

Third, we quantified the contribution of Hes1^CE^-E10.5, Hes1^CE^-E12.5, or Hes1^CE^-E14.5 cells to SOX9^+^ chondrocytes within the cartilage template. The chondrogenic potential was unique to Hes1^CE^-E10.5 cells. These cells contributed progressively to a larger fraction of SOX9^+^ chondrocytes within the growth plate during the chase (Fig. 3*I*, upper right panel).

Therefore, Hes1^CE^-E10.5 peri-condensation mesenchymal cells have both chondrogenic and osteogenic potentials, while Hes1^CE^-E12.5 perichondrial cells have only osteogenic potentials. Importantly, Hes1^+^ descendants appeared to continue expressing *Hes1* in the perichondrium, as the relative fraction of Hes1^CE^-E10.5 and Hes1^CE^-E12.5 cells in the perichondrium remained comparable at E13.5. Interestingly Hes1^CE^-E14.5 cells lose their robust potential to contribute neither chondrocyte or osteoblast, indicating that the ability of Hes1^+^ cells to generate chondrocytes and osteoblasts is restricted to an early stage of endochondral bone development.

### Hes1-creER^+^ peri-condensation mesenchymal cells contribute to postnatal marrow stroma *via* cartilage-dependent and independent pathways

**Figure 4:**
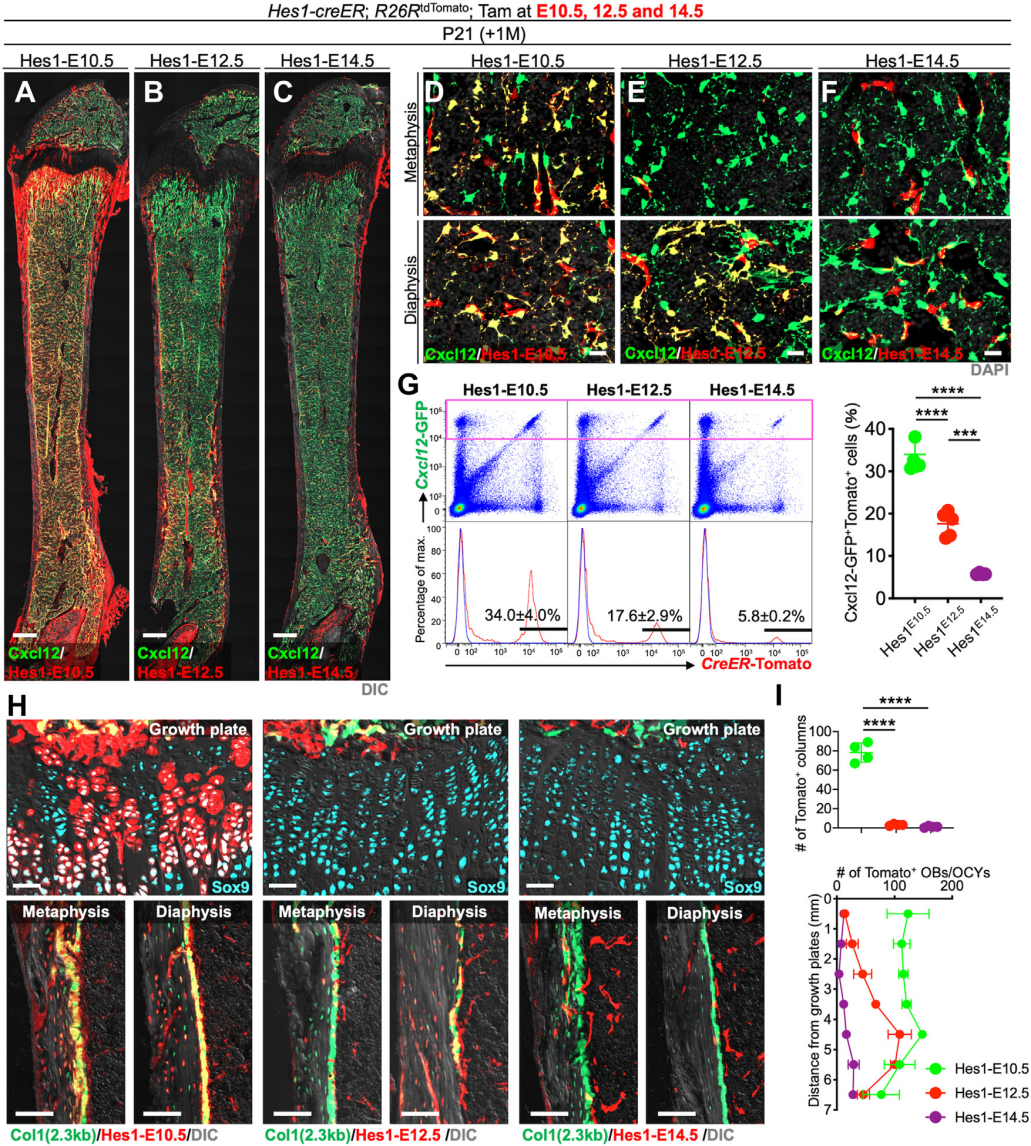
**Early developmental Hes1-creER**^**+**^**cells outside the Sox9**^**+**^**cells contribute to the postnatal skeletal compartment in a time-dependent manner.***A*–*G*, Contribution of fetal *Hes1-creER*^+^ cells, pulsed at E10.5 (*A* and *D*), E12.5 (*B* and *E*), or E14.5 (*C* and *F*) to Cxcl12-GFP^+^ bone marrow stromal cells at P21. *Cxcl12*^*GFP/+*^; *Hes1-creER*; *R26R*^*tdTomato*^ femurs with growth plates on top. *A*–*C*, Whole bone, upper panels in (*D*–*F*): metaphyseal bone marrow, lower panels in (*D*–*F*) diaphyseal bone marrow. Scale bar: 500 μm (*A*–*C*), 20 μm (*D*–*F*). *n* = 4 mice per group. *G*, Percentage of Cxcl12-GFP^high^tdTomato^+^ cells per total Cxcl12-GFP^high^ cells. *n* = 4 mice per group. ∗∗∗*p* < 0.001, ∗∗∗∗*p* < 0.0001. Two-tailed, One-way ANOVA followed by Tukey’s post-hoc test (the others). Data are presented as mean ± s.d. *H* and *I*, Contribution of fetal *Hes1-creER*^+^ cells to growth plate chondrocytes and cortical osteoblasts and osteocytes at P21. *H*, *Col1a1(2.3kb)-GFP*; *Hes1-creER*; *R26R*^tdTomato^, pulsed at E10.5 (*left*), E12.5 (*center*) and E14.5 (*right*). Growth plate (*upper*), endocortical marrow space at metaphysis (*lower left*) and diaphysis (*lower right*). Scale bar: 50 μm (*upper panels*), 100 μm (l*ower panels*). *n* = 4 mice per each group. *I*, *upper*, quantification of tdTomato^+^ columns in growth plate. Lower, Quantification of tdTomato^+^ osteoblasts/cytes, based on the distance from growth plate. *Green*: Hes1-E10.5, *Red*: Hes1-E12.5, *Violet*: Hes1-E14.5. n = 4 mice per each group. ∗∗∗∗*p* < 0.0001. Two-tailed, One-way ANOVA followed by Tukey’s *post hoc* test (the others). Data are presented as mean ± SD.

We further set out to define how Hes1^+^ cells contribute to the postnatal bone marrow stromal compartment. For this purpose, we utilized *Hes1-creER*; *R26R*^*tdTomato*^ mice carrying *Cxcl12-GFP* reporter, and analyzed Hes1^CE^-E10.5, Hes1^CE^-E12.5, and Hes1^CE^-E14.5 cells at P21. Notably, Hes1^CE^-E10.5 cells contributed to the entire bone marrow compartment, generating Cxcl12-GFP^high^ stromal cells both in the metaphyseal and diaphyseal marrow space (Fig. 4, *A* and *D*). In contrast, Hes1^CE^-E12.5 cells contributed only to Cxcl12-GFP^high^ stromal cells in the diaphyseal bone marrow stromal compartment (Fig. 4, *B* and *E*). Interestingly, Hes1^CE^-E14.5 cells contributed to only a small number of Cxcl12-GFP^high^ stromal cells; instead, these cells appeared to contribute to sinusoidal endothelial cells immediately adjacent to Cxcl12-GFP^high^ stromal cells (Fig. 4, *C* and *F*).

We quantitatively assessed how Hes1^+^ cells at different stages contribute differentially to Cxcl12-GFP^high^ stromal cells using flow cytometry analysis, using bone marrow cells isolated from P21 femurs of *Cxcl12*^*GFP/+*^*; Hes1-creER; R26R*^*tdTomato*^ triple transgenic mice pulsed at E10.5, E12.5 or E14.5 (Figs. 4*G* and S6*A*). Hes1^+^ peri-condensation mesenchymal cells at E10.5 demonstrated a robust potential to generate marrow stromal cells, as Hes1^CE^-E10.5 cells contributed to a substantial fraction of Cxcl12-GFP^high^ stromal cells (34.0 ± 4.0%) (Fig. 4*G*, right panel). In contrast, Hes1^+^ perichondrial cells at E12.5 contributed to a sizable but significantly smaller fraction of Cxcl12-GFP^high^ stromal cells (17.6 ± 2.9%). Hes1^CE^-E14.5 cells contributed to a much smaller fraction of Cxcl12-GFP^high^ stromal cells than Hes1^CE^-E10.5 and Hes1^CE^-E12.5 cells did (5.8 ± 0.2%), indicating that Hes1^+^ cells progressively lose their potential to generate bone marrow stromal cells in later stages.

We further quantified the contribution of Hes1^CE^-E10.5, Hes1^CE^-E12.5, and Hes1^CE^-E14.5 cells to postnatal growth plate chondrocytes and osteoblasts using histological approaches (Fig. 4*H*). Notably, Hes1^CE^-E10.5 cells, but not Hes1^CE^-E12.5 or Hes1^CE^-E14.5 cells, generated postnatal growth plate chondrocytes (Fig. 4*I*). Further, Hes1^CE^-E10.5 cells contributed robustly to osteoblasts/cytes both in the metaphysis and the diaphysis, while Hes1^CE^-E12.5 cells predominantly contributed to osteoblasts/cytes in the diaphysis. Hes1^CE^-E14.5 cells made only a small contribution to osteoblasts/cytes in the diaphysis (Figs. 4, *G* and *I* and S6*B*). Taken together, these findings indicate that Hes1^+^ peri-condensation mesenchymal cells generate postnatal marrow stroma *via* cartilage-dependent and independent pathways.

### Hes1-creER^+^ peri-condensation mesenchymal cells contribute to bone marrow colony forming fibroblasts

**Figure 5:**
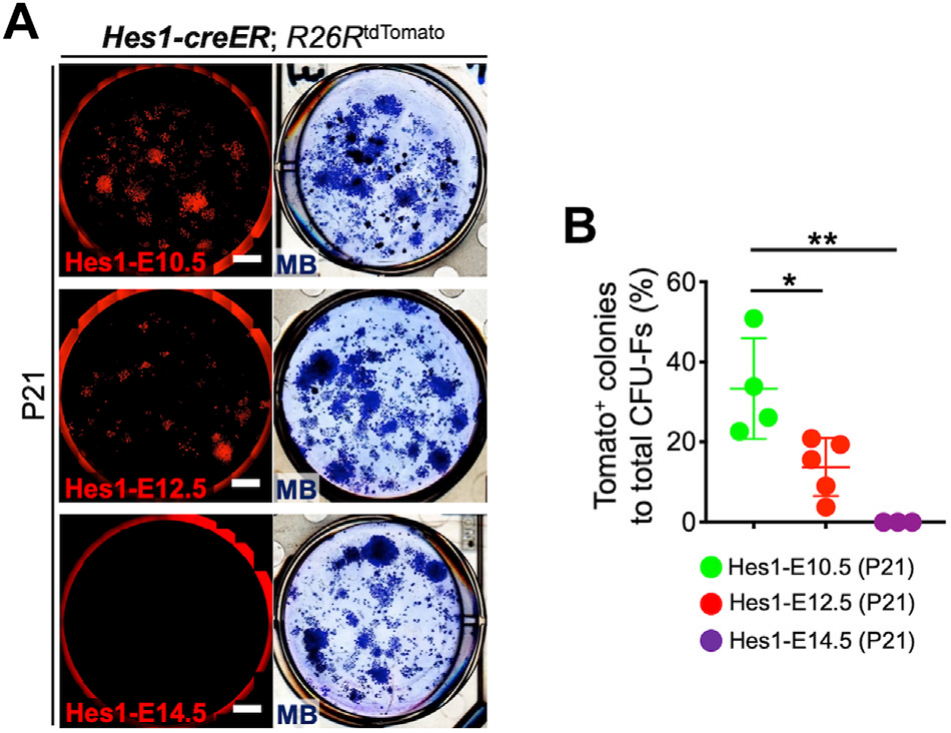
**Early developmental Hes1-creER**^**+**^**cell-derived marrow stromal cells behave as skeletal progenitor cells.***A*, Colony-forming unit fibroblast (CFU-F) assay of *Hes1-creER*; *R26R*^tdTomato^ at P21, pulsed at E10.5 (*top*), E12.5 (*middle*), and E14.5 (*bottom*). Scale bar: 5 mm. *B*, Percentage of tdTomato^+^ colonies among total CFU-Fs. *n* = 4 (Hes1-E12.5), *n* = 5 (Hes1-E12.5), *n* = 3 (Hes1-E14.5) mice. ∗*p* < 0.05, ∗∗*p* < 0.01. Two-tailed, One-way ANOVA followed by Tukey’s post-hoc test. Data are presented as mean ± SD. MB, Methylene blue staining.

Lastly, we asked whether Hes1^+^ peri-condensation mesenchymal cells can contribute to a fraction of putative skeletal stem cells in the bone marrow. To this end, we performed colony-forming unit fibroblast (CFU-F) assays of P21 bone marrow cells isolated from *Hes1-creER; R26R*^*tdTomato*^ mice pulsed at E10.5, E12.5 or E14.5, to discern the contribution of Hes1^CE^-E10.5, Hes1^CE^-E12.5 and Hes1^CE^-E14.5 cells to the clonogenic fraction. Hes1^CE^-E10.5 cells contributed to a substantial faction of CFU-Fs (Hes1^CE^-E10.5: 33.4 ± 12.6% of total CFU-Fs), while Hes1^CE^-E12.5 cells contributed to a significantly smaller fraction of CFU-Fs (Hes1^CE^-E12.5: 13.7 ± 7.2% of total CFU-Fs). In contrast, Hes1^CE^-E14.5 cells made no contribution to CFU-Fs (Hes1^CE^-E14.5: 0.0 ± 0.0% of total CFU-Fs, Fig. 5, *A* and *B*). Therefore, Hes1^+^ peri-condensation mesenchymal cells contribute to the clonogenic fraction of the postnatal bone marrow stroma, which may include a population of putative skeletal stem cells.

## Discussion

Here, we demonstrate that a population of early mesenchymal cells defined by Notch effector *Hes1* expression outside the SOX9^+^ condensation can provide an important source of chondrocytes and perichondrial cells in early endochondral bone development. These Hes1^+^ peri-condensation mesenchymal cells represent precursors of Dlx5^+^ early perichondrial cells that we reported recently (4) and robustly participate in chondrogenesis, osteogenesis, and subsequently the formation of the postnatal bone marrow stromal compartment through two routes of cartilage-dependent and independent pathways. Our *Hes1-creER*-based *in vivo* lineage-tracing findings establish the concept that SOX9-negative Hes1-expressing mesenchymal cells surrounding mesenchymal condensations play important roles in early endochondral bone development. Importantly, the robust chondrogenic and osteogenic potential of Hes1^+^ cells is unique to the mesenchymal condensation stage, whereas these cells progressively lose such potential in later stages.

We believe that the two lineages of chondrocytes and perichondrial cells diverge prior to E12.5, as Hes1^CE^-E10.5 cells, but not Hes1^CE^-E12.5 cells, contribute to chondrocytes within the cartilage template. Hes1^+^ cells at E10.5 and earlier time are likely to provide a common progenitor population for at least some of the chondrocytes and osteoblasts. We acknowledge, however, that our approach based on a single-color reporter cannot determine if an individual peri-condensation Hes1^+^ cell can contribute clonally both to chondrocytes and osteoblasts. An *in vivo* clonal analysis with a multi-color lineage reporter would facilitate the identification of a common progenitor population for chondrocytes and osteoblasts in the peri-condensation region.

Hes1 is one of the canonical Notch effector genes that is expressed by cells in which Notch signaling is activated. In fact, we observed that a Notch signaling reporter CBF1:H2B-Venus was specifically active in peri-condensation mesenchymal cells surrounding the SOX9^+^ pre-cartilaginous condensation. Notch signaling, which is primarily mediated by the CBF1-dependent target gene *Hes1*, regulates both *Sox9* and *Runx2* expression during skeletal cell differentiation (22). Hes1 inhibits *Sox9* expression, and Hes1 inhibition promotes chondrogenesis by upregulating *Sox9*. Hes1 also inhibits osteoblast differentiation through the inhibition of Runx2 activities (23). Notch signaling regulates asymmetric cell division and lineage decision through lateral inhibition. It is possible that Hes1^+^ peri-condensation mesenchymal cells divide into Hes1-positive and negative cells through asymmetric cell division; the former Hes1-positive cells may stay in the perichondrium as undifferentiated skeletal progenitors, while the latter Hes1-negative cells enter into the cartilage template and differentiate into chondrocytes.

We show that *Hes1-creER* allows the marking of early mesenchymal cells in a SOX9-negative domain of the condensation. Historically, *Prrx1-cre/creER* has been widely utilized as a tool to mark “*skeletal stem/progenitor cells*” at the condensation stage (13, 26). However, the caveat is that *Prrx1-cre* simultaneously marks SOX9^+^ cells within the condensation, making it impossible to discern the contribution of SOX9-negative peri-condensation mesenchymal cells. Our findings from *Hes1-creER*-based lineage-tracing experiments revise the concept regarding the origin of osteo-chondroprogenitor cells within the limb bud, which is currently considered to rest solely upon SOX9^+^ (Sox9-cre^+^) cells (9). The emerging concept is that both SOX9^+^ cells within the condensation and SOX9-negative cells outside the condensation play equally important roles in early endochondral bone development. Identifying the unique role of each respective cell-of-origin of the limb bud mesenchyme remain as an important agenda for future studies.

**Figure 6:**
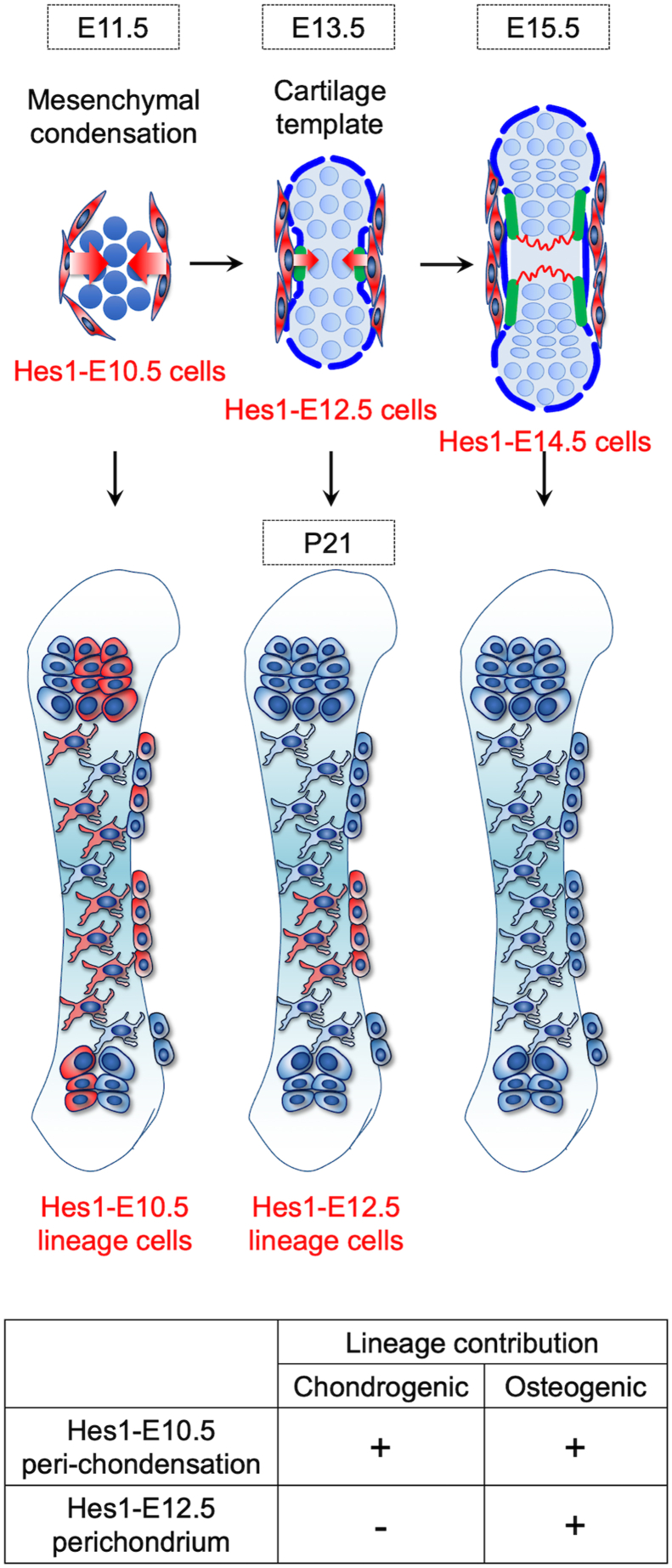
**Hes1-marked early mesenchymal cells surrounding the condensation behave as skeletal progenitors in endochondral bone development.** Early perichondrial population of skeletal progenitor cells in endochondral bone development. Undifferentiated mesenchymal cells surrounding condensation or outer perichondrial cells outside the cartilage template provide an important source during fetal endochondral bone development. During the condensation stage, Hes1^+^ undifferentiated mesenchymal cells take two distinct routes to participate in endochondral bone development. First, these cells translocate into the cartilage template and directly differentiate into Sox9^+^ chondrocytes and then contribute to bone formation (chondrocyte-dependent pathway). Second, these cells contribute to the outer layer of the perichondrium and become Hes1+ perichondrial cells. These Hes1+ perichondrial cells translocate into the nascent marrow space and directly differentiate into marrow mesenchymal cells by bypassing a Sox9+ state (chondrocyte-independent pathway). These perichondrial skeletal progenitor cells keep providing osteoblasts and bone marrow stromal cells even during the postnatal stage. As a result, postnatal bone marrow is characterized by transitional mosaicism composed of both chondrocyte-derived and perichondrium-derived stromal cells.

In conclusion, we propose a new concept that peri-condensation mesenchymal cells surrounding the SOX9^+^ condensation provide an important source of skeletal progenitor cells in early endochondral bone development (see the proposed diagram in Fig. 6).

## Experimental procedures

### Mouse strains

*Hes1-creER* (Hes1^tm1(cre/ERT2)Lcm^) (25) and *Cxcl12*^*GFP/+*^ (Cxcl12^tm2Tng^) (27) mice have been described previously. *Prrx1-cre* (JAX005584), *Acta1-cre* (JAX006139), *Myl-cre* (JAX024713), *Mck-cre* (JAX006475), *Tie2-cre* (JAX008863), *Rosa26-CAG-loxP-stop-loxP-tdTomato* (Ai14: *R26R-tdTomato*, JAX007914), *Rosa26-SA-loxP-GFP-stop-loxP-DTA* (JAX006331), *Col1a1(2.3kb)-GFP* (JAX013134), and *CBF:H2B-Venus* (JAX020942) mice were acquired from the Jackson laboratory. All procedures were conducted in compliance with the Guidelines for the Care and Use of Laboratory Animals approved by the University of Texas Health Science Center at Houston’s Animal Welfare Committee (AWC), protocol AWC-21-0070, and the University of Michigan’s Institutional Animal Care and Use Committee (IACUC), protocol 9496. All mice were housed in a specific pathogen-free condition and analyzed in a mixed background. Mice were housed in static microisolator cages (Allentown Caging). Access to water and food (irradiated LabDiet 5008) was ad libitum. Animal rooms were climate controlled to provide temperatures of 22 to 23 °C, 40 to 65% of humidity on a 12 h light/dark cycle (lights on at 0600). For all breeding experiments, *creER* transgenes were maintained in male breeders to avoid spontaneous germline recombination. Mice were identified by micro-tattooing or ear tags. Tail biopsies of mice were lysed by a HotShot protocol (incubating the tail sample at 95^o^C for 30 min in an alkaline lysis reagent followed by neutralization) and used for PCR-based genotyping (GoTaq Green Master Mix, Promega, and Nexus X2, Eppendorf). Perinatal mice were also genotyped fluorescently (BLS miner's lamp) whenever possible. Mice were euthanized by over-dosage of carbon dioxide or decapitation under inhalation anesthesia in a drop jar (Fluriso, Isoflurane USP, VetOne).

### Tamoxifen and induction of *cre-loxP* recombination

Tamoxifen (Sigma T5648) was mixed with 100% ethanol until completely dissolved. Subsequently, a proper volume of sunflower seed oil (Sigma S5007) was added to the tamoxifen-ethanol mixture and rigorously mixed. The tamoxifen-ethanol-oil mixture was incubated at 60 ^°^C in a chemical hood until the ethanol evaporated completely. The tamoxifen-oil mixture was stored at room temperature until use. Tamoxifen was injected at a dose of 3 mg into pregnant mice intraperitoneally using a 26 to 1/2-gauge needle (BD309597).

### Histology and immunohistochemistry

Samples were dissected under a stereomicroscope (Nikon SMZ-800), and fixed in 4% paraformaldehyde for a proper period, typically ranging from 3 h to overnight at 4^o^C, then decalcified in 15% EDTA for a proper period, typically ranging from 3 h to 14 days. Embryonic samples were not decalcified. Subsequently, samples were cryoprotected in 30% sucrose/PBS solutions and then in 30% sucrose/PBS:OCT (1:1) solutions, each at least overnight at 4 ^°^C. Samples were embedded in an OCT compound (Tissue-Tek, Sakura) under a stereomicroscope and transferred on a sheet of dry ice to solidify the compound. Embedded samples were cryosectioned at 14 μm using a cryostat (Leica CM1850) and adhered to positively charged glass slides (Fisherbrand ColorFrost Plus). Sections were postfixed in 4% paraformaldehyde for 15 min at room temperature. For immunostaining, sections were permeabilized with 0.25% TritonX/TBS for 30 min, blocked with 3% BSA/TBST for 30 min and incubated with rabbit anti-Sox9 polyclonal antibody (1:500, EMD-Millipore, AB5535), rat anti-endomucin (Emcn) monoclonal antibody (1:100, Santa Cruz Biotechnology, sc65495), rabbit anti-Myh3 polyclonal antibody (1:500, Abcam, ab124205),or rabbit anti-Osx polyclonal antibody (1:500, Abcam, ab22552) overnight at 4 ^°^C, and subsequently with Alexa Fluor 647-conjugated donkey anti-rabbit IgG (A31573) or Alexa Fluor 633-conjugated goat anti-rat IgG (A21049) (1:400, Invitrogen) for 3 h at room temperature. Sections were further incubated with DAPI (4′,6-diamidino-2-phenylindole, 5 μg/ml, Invitrogen D1306) to stain nuclei prior to imaging.

### RNAscope *in situ* hybridization

Samples were fixed in 4% paraformaldehyde overnight at 4 ^°^C and then cryoprotected. Frozen sections at 14 μm were prepared on positively charged glass slides. *In situ* hybridization was performed with RNAscope 2.5 HD Reagent kit Brown (Advanced Cell Diagnostics 322,300) using the following probes: *Hes1* (417701) and *Sox9* (custom-designed) according to the manufacturer’s protocol.

### Imaging and cell quantification

Images were captured by an automated inverted fluorescence microscope with a structured illumination system (Zeiss Axio Observer Z1 with ApoTome.2 system) and Zen 2 (blue edition) software. The filter settings used were: FL Filter Set 34 (Ex. 390/22, Em. 460/50 nm), Set 38 HE (Ex. 470/40, Em. 525/50 nm), Set 43 HE (Ex. 550/25, Em. 605/70 nm), Set 50 (Ex. 640/30, Em. 690/50 nm), and Set 63 HE (Ex. 572/25, Em. 629/62 nm). The objectives used were: Fluar 2.5x/0.12, EC Plan-Neofluar 5x/0.16, Plan-Apochromat 10x/0.45, EC Plan-Neofluar 20x/0.50, EC Plan-Neofluar 40x/0.75, Plan-Apochromat 63x/1.40. Images were typically tile-scanned with a motorized stage, Z-stacked, and reconstructed by a maximum intensity projection (MIP) function. Differential interference contrast (DIC) was used for objectives higher than 10×. Representative images of at least three independent biological samples are shown in the figures. Quantification of cells on sections was performed using NIH Image J software.

### Cell preparation

For embryonic samples, hind limbs were harvested and incubated with 2 Wunsch units of Liberase TM (Sigma/Roche 5401127001) in 2 ml Ca^2+^, Mg^2+^-free Hank’s Balanced Salt Solution (HBSS, Sigma H6648) at 37 ^°^C for 15 min on a shaking incubator (ThermomixerR, Eppendorf). For postnatal samples, soft tissues and epiphyses were carefully removed from dissected femurs. After removing distal epiphyseal growth plates and cutting off proximal ends, femurs were cut roughly and incubated with 2 Wunsch units of Liberase TM and 1 mg of Pronase (Sigma/Roche 10165921001) in 2 ml Ca^2+^, Mg^2+^-free HBSS at 37 ^°^C for 60 min on a shaking incubator. After cell dissociation, cells were mechanically triturated using an 18-gauge needle with a 1 ml Luer-Lok syringe (BD) and a pestle with a mortar (Coors Tek), and subsequently filtered through a 70 μm cell strainer (BD) into a 50 ml tube on ice to prepare single cell suspension. These steps were repeated for five times, and dissociated cells were collected in the same tube. Cells were pelleted and resuspended in an appropriate medium for subsequent purposes. For cell culture experiments, cells were resuspended in 10 ml culture medium and counted on a hemocytometer.

### Flow cytometry

Dissociated cells were stained by standard protocols with the following antibodies (1:500, eBioscience). Allophycocyanin (APC)-conjugated CD31 (390, endothelial/platelet), CD45 (30F-11, hematopoietic), and Ter119 (TER-119, erythrocytes). Flow cytometry analysis was performed using a four-laser BD LSR Fortessa (Ex. 405/488/561/640 nm) and FACSDiva software. Acquired raw data were further analyzed on FlowJo software (TreeStar). Representative plots of at least four independent biological samples are shown in the figures.

### Single-cell RNA-seq analysis of fluorescence-activated cell sorting-isolated cells

Cell sorting was performed using a four-laser BD fluorescence-activated cell sorting (FACS) Aria III (E x .407/488/561/640 nm) high-speed cell sorter with a 100 μm nozzle. tdTomato^+^ cells were directly sorted into ice-cold DPBS/1% BSA, pelleted by centrifugation and resuspended in appropriate amount of DPBS/1% BSA (1000 cells/μl). Cell numbers were quantified by Countless II automated Cell Counter (ThermoFisher) before loading onto the Chromium Single Cell 3′ v2 microfluidics chip (10x Genomics Inc). cDNA libraries were sequenced by Illumina HiSeq 4000 using two lanes and 50 cycle paired-end read, generating a total of ∼770 million reads. The sequencing data was first pre-processed using the 10X Genomics software Cell Ranger. For alignment purposes, we generated and used a custom genome fasta and index file by including the sequences of *tdTomato-WPRE* to the mouse genome (mm10). Further downstream analysis steps were performed using the Seurat (20) R package. We filtered out cells with less than 500 genes per cell and with more than 20% mitochondrial read content. The downstream analysis steps include normalization, identification of highly variable genes across the single cells, scaling based on number of UMI, dimensionality reduction (PCA, CCA, and t-SNE), unsupervised clustering, and the discovery of differentially expressed cell-type specific markers. Differential gene expression to identify cell-type specific genes was performed using the nonparametric Wilcoxon rank sum test.

### Colony-forming assay and subcloning

Nucleated bone marrow cells were plated into tissue culture 6-well plates (BD Falcon) at a density of <10^5^ cells/cm^2^ and cultured in low-glucose DMEM with GlutaMAX supplement (Gibco 10567022) and 10% mesenchymal stem cell-qualified FBS (Gibco 12662029) containing penicillin-streptomycin (Sigma P0781) for 10∼14 days. Cell cultures were maintained at 37 ^°^C in a 5% CO_2_ incubator. Representative images of at least three independent biological samples are shown in the figures. For CFU-Fs, cells were fixed with 70% Ethanol for 5 min and stained for 2% methylene blue.

### Statistical analysis

Results are presented as mean values ± SD. Statistical evaluation was conducted using the Mann-Whitney's *U*-test or one-way ANOVA. A *p* value of < 0.05 was considered significant. No statistical method was used to predetermine sample size. Sample size was determined on the basis of previous literature and our previous experience to give sufficient standard deviations of the mean so as not to miss a biologically important difference between groups. The experiments were not randomized. All of the available mice of the desired genotypes were used for experiments. The investigators were not blinded during experiments and outcome assessment. One femur from each mouse was arbitrarily chosen for histological analysis. Genotypes were not particularly highlighted during quantification.

## Data availability

The data generated during and/or analyzed during the current study are available from the corresponding author upon reasonable request. The single-cell RNA-seq data presented herein has been deposited in the National Center for Biotechnology Information (NCBI)’s Gene Expression Omnibus (GEO) and are accessible through GEO Series accession number GSE144411 (https://www.ncbi.nlm.nih.gov/geo/query/acc.cgi?acc=GSE144411).

## Supporting information

This article contains supporting information.

## Conflict of interest

The authors declare that they have no known competing financial interests or personal relationships that could have appeared to influence the work reported in this paper.
