# Supplementary material for: Hes1 marks peri-condensation mesenchymal cells that generate both chondrocytes and perichondrial cells in early bone development
Source: J Biol Chem. 2023 May 11;299(6):104805. doi: 10.1016/j.jbc.2023.104805 (PMC10267520; doi:10.1016/j.jbc.2023.104805)
Supplement: Supporting Figures S1–S6 [file mmc1.pdf]

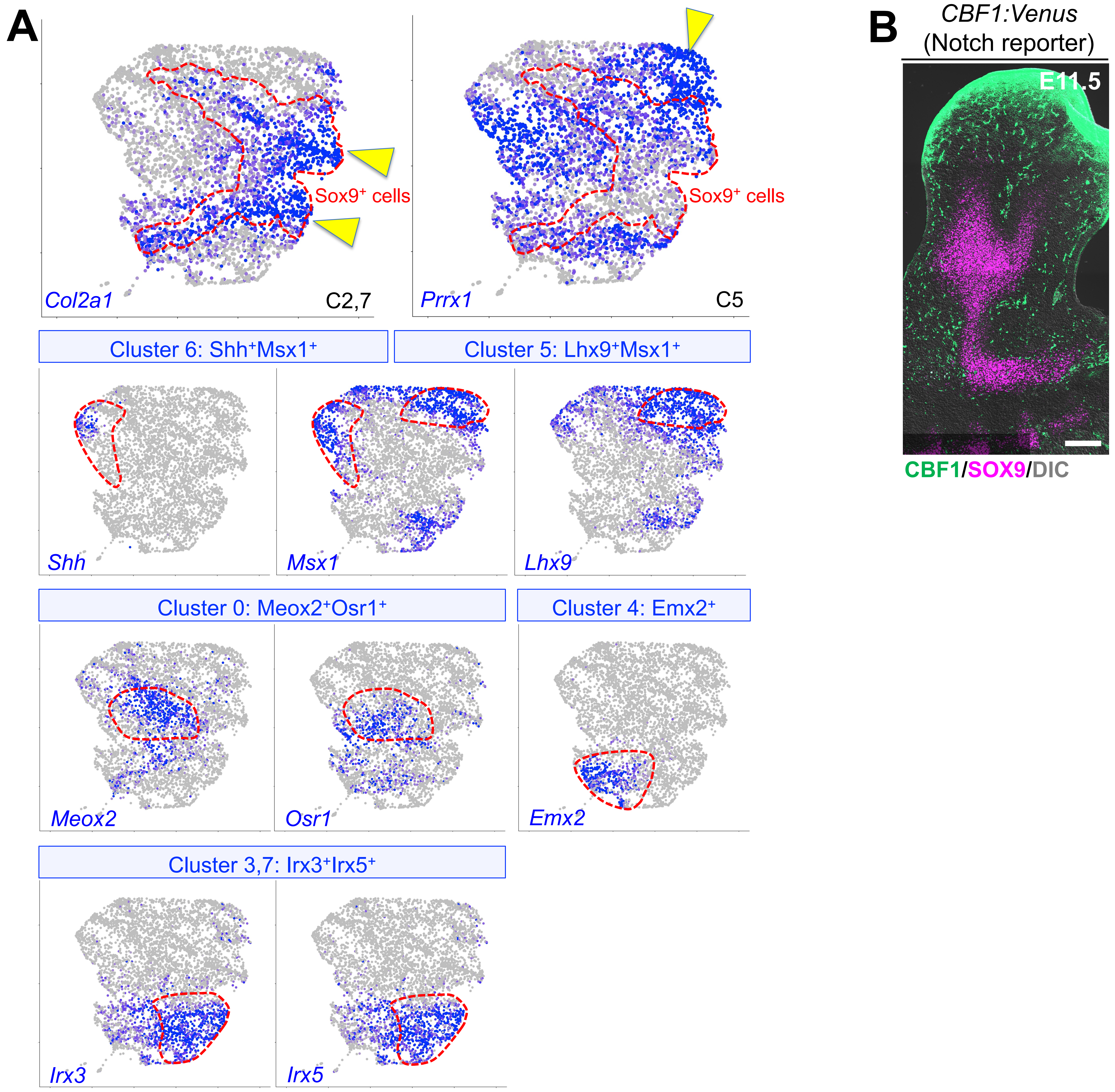


**Supplementary Figure 1. Single-cell RNA-seq identifies *Hes1* as a potential marker of peri-condensation mesenchymal cells.**

**(A)** Feature plots of genes enriched in each cluster from *Prrx1-cre*-marked limb bud mesenchymal cell-based single cell RNA-seq analysis at E11.5. Cluster 2,7: *Col2a1*$^+$, Cluster 5: *Prrx1*$^+$, Cluster 6: *Shh*$^+$*Msx1*$^+$, Cluster 5: *Lhx9*$^+$*Msx1*$^+$, Cluster 0: *Meox2*$^+$*Osr1*$^+$, Cluster 4: *Emx2*$^+$, Cluster 3,7: *Irx3*$^+$*Irx5*$^+$. Red contour: featured clusters. **(B)** Notch reporter *CBF1:H2B-Venus* femur immunostained for SOX9 at E11.5. Scale bar: 200µm. *n*=3 mice.

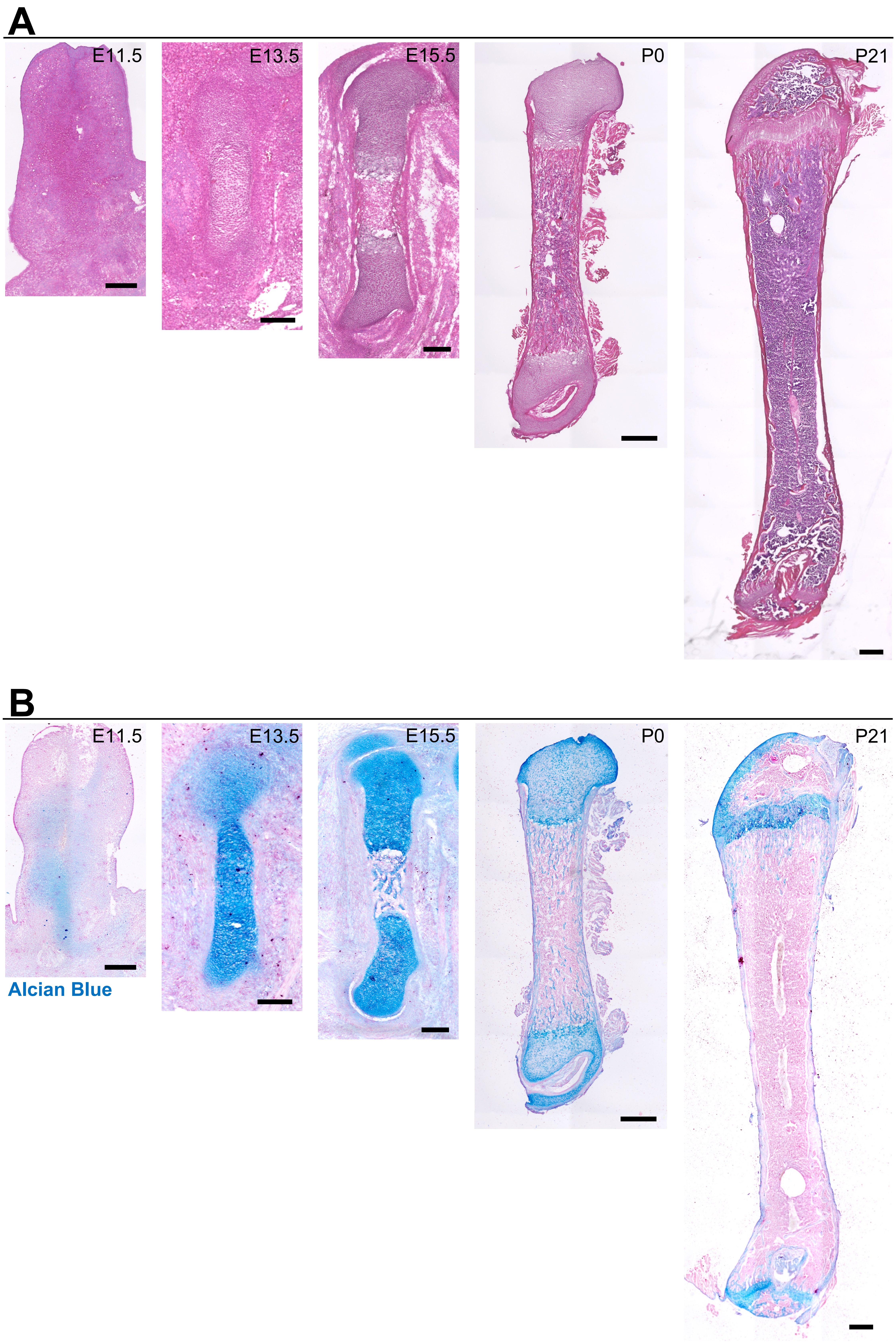


**Supplementary Figure 2. Stages of endochondral bone development.**

Sequential steps of endochondral bone development. (A): H&E staining, (B): Alcian Blue staining. E11.5: mesenchymal condensation. E13.5: cartilage template and perichondrium. E15.5: formation of the primary ossification center and incipient bone marrow. P0: at birth. P21: at weaning. Scale bar: 200µm (E11.5, E13.5, and E15.5). 500µm (P0 and P21). *n*=4 mice.

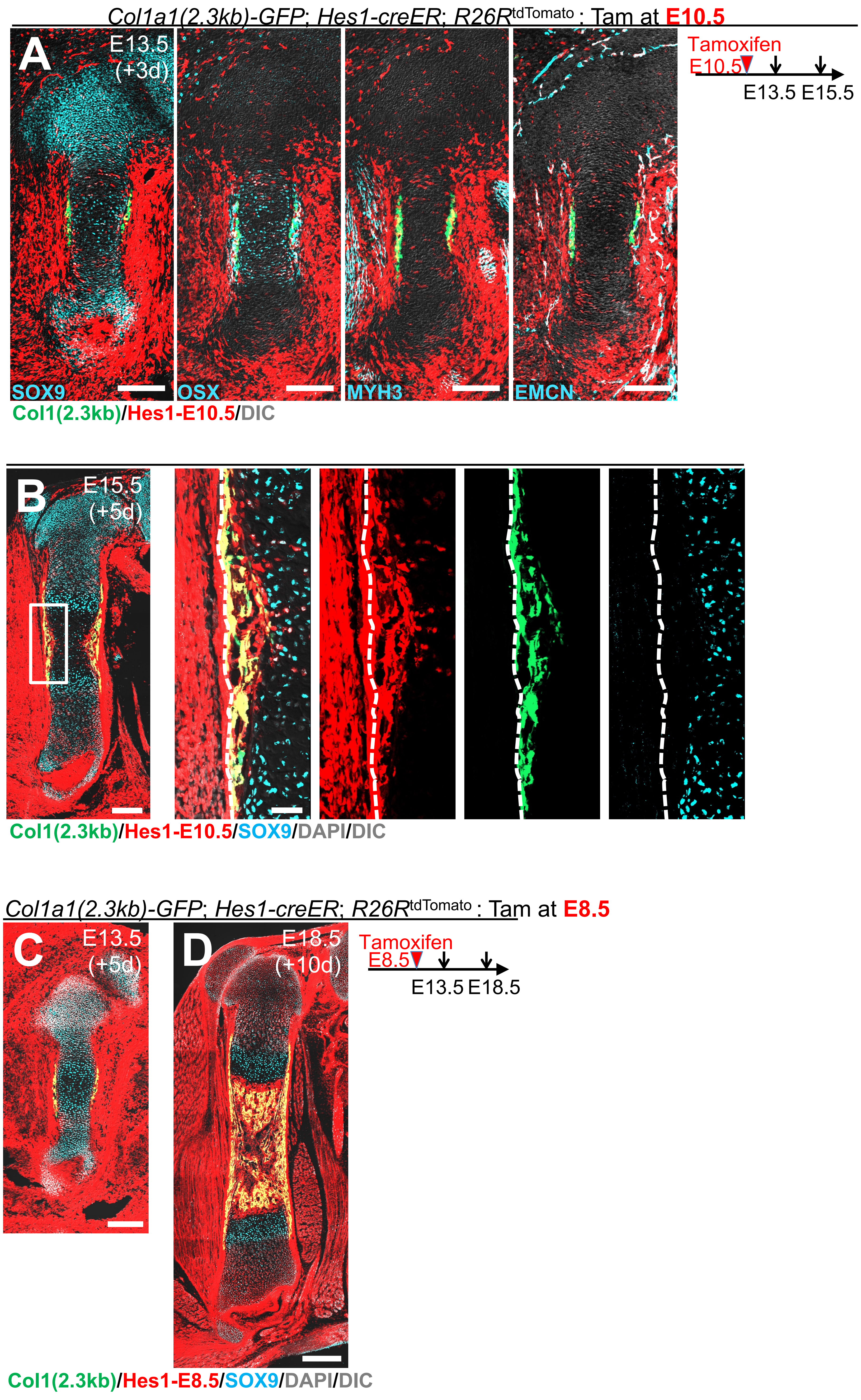


**Supplementary Figure 3. Hes1-creER⁺ peri-condensation mesenchymal cells can generate chondrocytes.**

**(A)** Cartilage template at E13.5, after 3 days of chase (pulsed at E10.5). Immunostaining for SOX9, OSX, MYH3 and EMCN. Scale bar: 200μm. *n*=4 mice.
**(B)** Embryonic femur at E15.5, after 5 days of chase (pulsed at E10.5). Immunostaining for SOX9. Scale bar: 200μm (left), 50μm (right 4 panels). *n*=4 mice.
**(C)** Cartilage template at E13.5, after 5 days of chase (pulsed at E8.5). Immunostaining for SOX9. Scale bar: 200μm. *n*=4 mice.
**(D)** Embryonic femur at E18.5, after 10 days of chase (pulsed at E8.5). Immunostaining for SOX9. Scale bar: 500μm. *n*=4 mice.

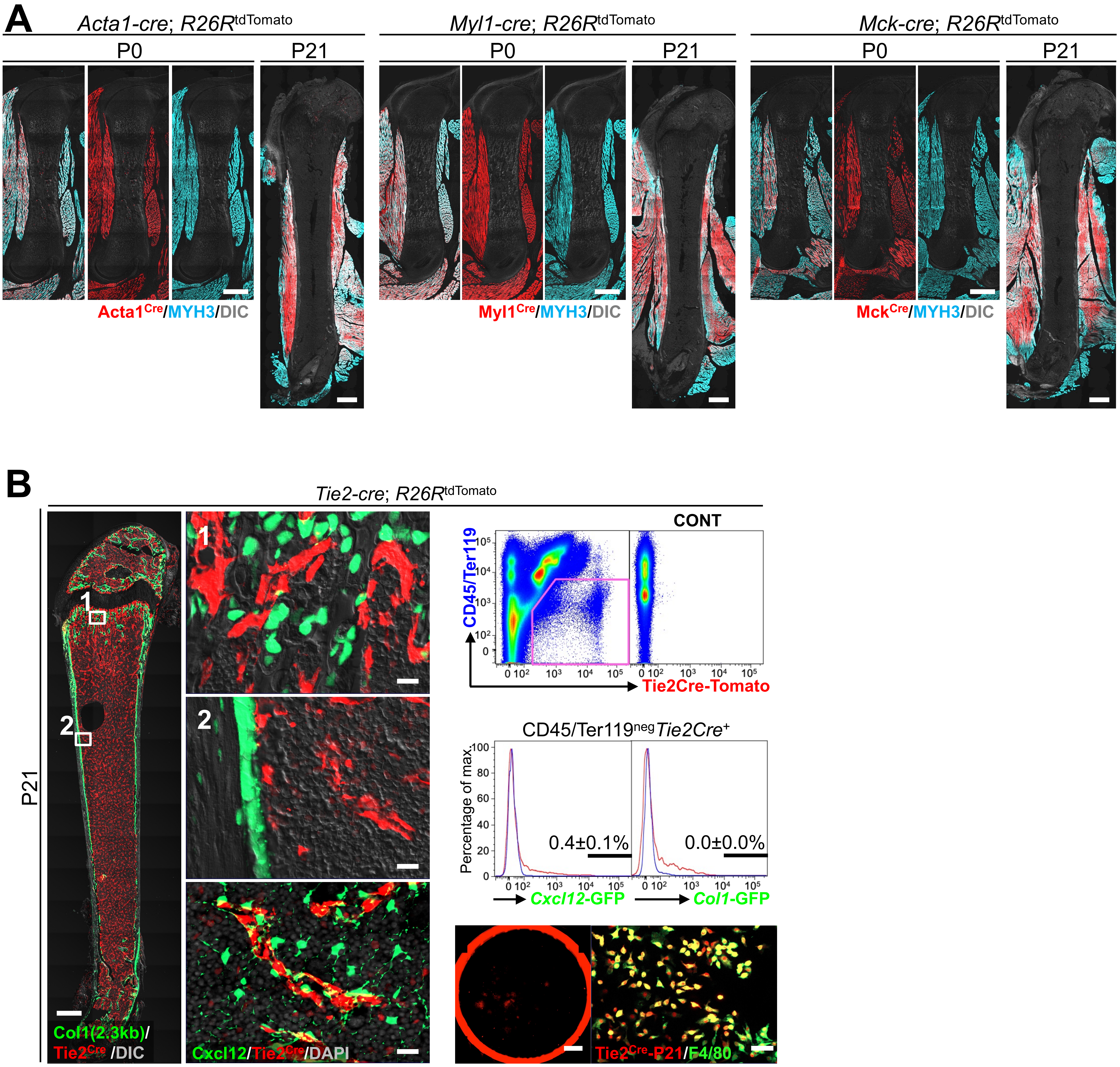


**Supplementary Figure 4. Skeletal muscle cells or endothelial cells do not contribute to chondrocytes, osteoblasts or bone marrow stromal cells.**

**(A)** Fate mapping analysis of *Acta1-cre, Myl1-cre or Mck-cre*-marked skeletal muscle cells at P0 (top) and P21 (bottom). *Acta1-cre*; $R26R^{tdTomato}$ (left), *Myl1-cre*; $R26R^{tdTomato}$ (center) and *Mck-cre*; $R26R^{tdTomato}$ (right) femurs. Immunostaining for MYH3. Grey: DIC. Scale bar: 500µm. *n*=3 mice per each group.

**(B)** Fate-mapping analysis of *Tie2-cre*-marked endothelial cells at P21. *Col1a1(2.3kb)-GFP*; *Tie2-cre*; $R26R^{tdTomato}$ and $Cxcl12^{GFP/+}$; *Tie2-cre*; $R26R^{tdTomato}$ femurs. Left panel: whole bone. Scale bar: 500µm. Upper center panels: magnified views of boxed areas (1: trabecular bone, 2: endosteal marrow space). Scale bar: 20µm. Lower center panel: magnified view of diaphyseal bone marrow of $Cxcl12^{GFP/+}$; *Tie2-cre*; $R26R^{tdTomato}$ femur. Scale bar: 20µm. *n*=3 mice per each group. Upper right panels: flow cytometry analysis of bone marrow cells. Right center panel: histogram showing GFP expression. Blue lines: control cells. *n*=5 ($Cxcl12^{GFP/+}$), *n*=4 (*Col1a1(2.3kb)-GFP*) mice. Lower right panels: CFU-F assay of *Tie2-cre*; $R26R^{tdTomato}$ bone marrow cells. Left: tdTomato epifluorescence. Scale bar: 5mm. Right: F4/80 immunostaining. Scale bar: 50µm. *n*=3 mice.

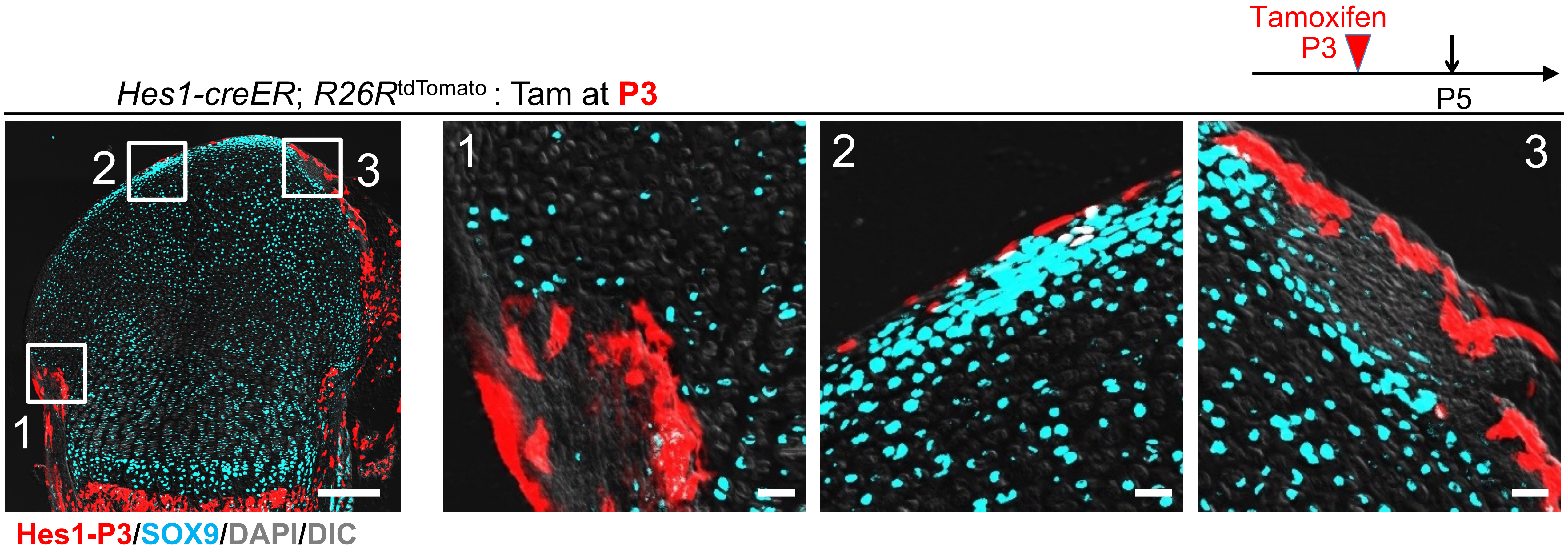


**Supplementary Figure 5. Hes1-creER+ cells at an early postnatal stage.**

Cell-fate analysis of *Hes1-creER*+ cells. Distal area of femur at P5 (pulsed at P3). Immunostaining for SOX9. 1: Groove of Ranvier. 2 and 3: Articular cartilage. Scale bar: 500µm (left), 50µm (right 3 panels). *n*=4 mice.

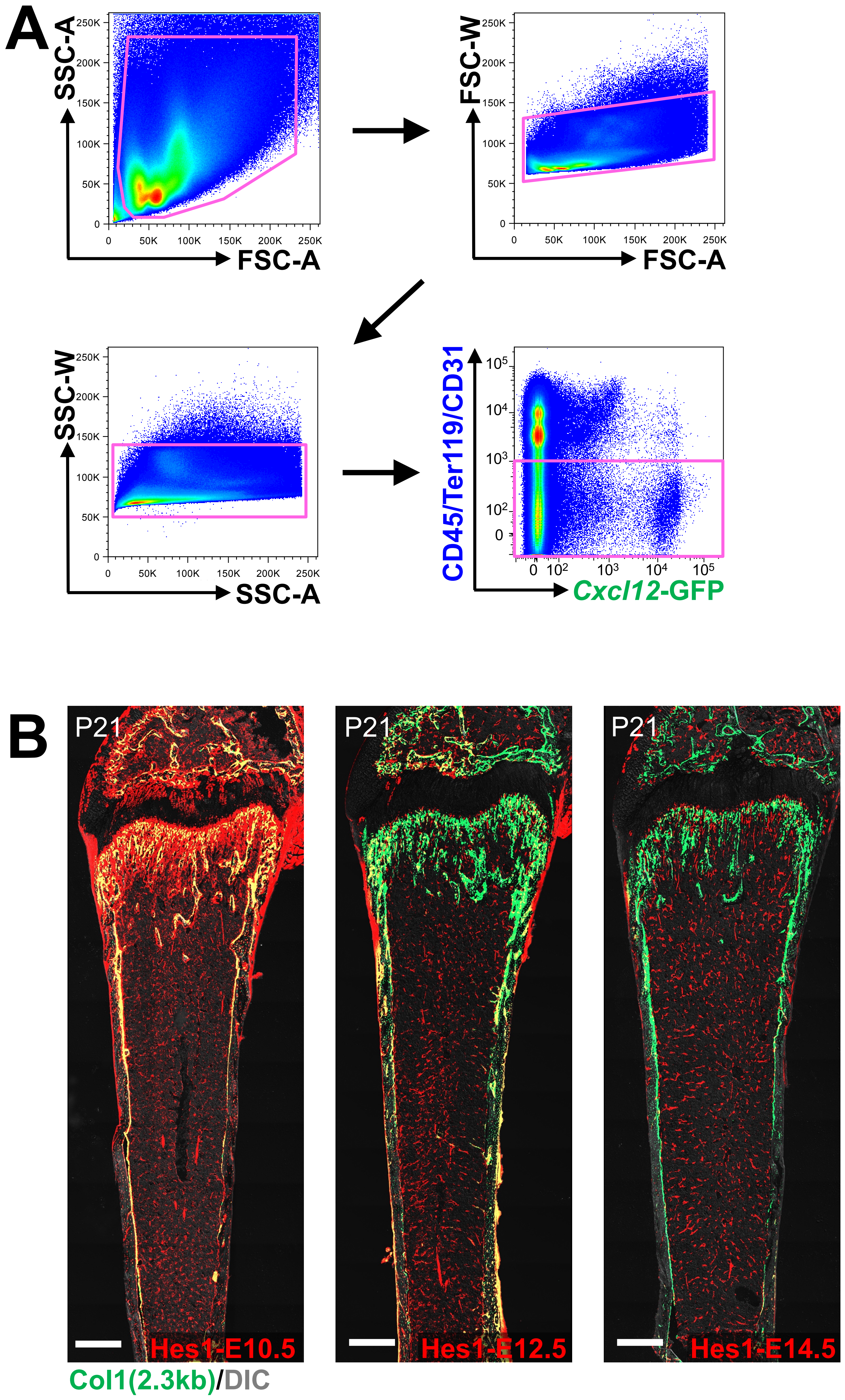


**Supplementary Figure 6. Contribution of Hes1-creER$^{+}$ cells to the postnatal stromal compartment**

**(A)** Flow cytometry analysis of CD45/Ter119/ CD31$^{neg}$ cells at P21. Gating strategy for bone marrow cells isolated from *Cxcl12*$^{GFP/+}$; *Hes1-creER*; *R26R*$^{tdTomato}$ femurs (pulsed at E10.5, E12.5 or E14.5). **(B)** Contribution of fetal *Hes1-creER*$^{+}$ cells (pulsed at E10.5, E12.5 or E14.5) to skeleton at P21. *Col1a1(2.3kb)-GFP*; *Hes1-creER*; *R26R*$^{tdTomato}$ femurs with growth plates on top. Scale bar: 500µm (left panels). *n*=4 mice per each group.
